# Supervised training in primary care units but not self-directed physical activity lowered cardiovascular risk in Brazilian low-income patients: a controlled trial

**DOI:** 10.1186/s12889-019-7716-y

**Published:** 2019-12-27

**Authors:** Amana M. Lima, André O. Werneck, Edilson Cyrino, Paulo Farinatti

**Affiliations:** 1 Graduate Program in Physical Activity Sciences, Salgado de Oliveira University, Rua Marechal Deodoro 217, Bloco C (Anexo), 2° Andar, Centro, Niteroi, RJ 24030-060 Brazil; 2 Laboratory of Investigation in Exercise (LIVE), Department of Physical Education, São Paulo State University (UNESP), Presidente Prudente, SP Brazil; 3 Study and Research Group in Metabolism, Nutrition, and Exercise (GEPEMENE), Londrina State University, Londrina, PR Brazil; 4 Laboratory of Physical Activity and Health Promotion (LABSAU), University of Rio de Janeiro State, Rio de Janeiro, RJ Brazil

**Keywords:** Cardiovascular health, Exercise training, Health promotion, Physical education, Public health, Quasi-experimental trial

## Abstract

**Background:**

Public health strategies to increase physical activity in low-income communities may reduce cardiovascular risk in these populations. This controlled trial compared the cardiovascular risk estimated by the Framingham Risk Score (FRS) over 12 months in formally active (FA), declared active (DA), and physically inactive (PI) patients attended by the ‘Family Health Strategy’ in low-income communities at Rio de Janeiro City, Brazil (known as *‘favelas’*).

**Methods:**

Patients were matched for age and assigned into three groups: a) FA (supervised training, *n* = 53; 60.5 ± 7.7 yrs); b) DA (self-reported, *n* = 43; 57.0 ± 11.2 yrs); c) PI (*n* = 48; 57.0 ± 10.7 yrs). FA performed twice a week a 50-min exercise circuit including strength and aerobic exercises, complemented with 30-min brisk walking on the third day, whereas DA declared to perform self-directed physical activity twice a week. Comparisons were adjusted by sex, chronological age, body mass index, and use of anti-hypertensive/statin medications.

**Results:**

At baseline, groups were similar in regards to body mass, body mass index, triglycerides, and LDL-C, as well to FRS and most of its components (age, blood pressure, hypertension prevalence, smoking, HDL-C, and total cholesterol; *P* > 0.05). However, diabetes prevalence was 10–15% lower in DA vs. FA and PI (*P* < 0.05). Intention-to-treat analysis showed significant reductions after intervention (*P* < 0.05) in FA for total cholesterol (~ 10%), LDL-C (~ 15%), triglycerides (~ 10%), systolic blood pressure (~ 8%), and diastolic blood pressure (~ 9%). In DA, only LDL-C decreased (~ 10%, *P* < 0.05). Significant increases were found in PI (*P* < 0.05) for total cholesterol (~ 15%), LDL-C (~ 12%), triglycerides (~ 15%), and systolic blood pressure (~ 5%). FRS lowered 35% in FA (intention-to-treat, *P* < 0.05), remained stable in DA (*P* > 0.05), and increased by 20% in PI (*P* < 0.05).

**Conclusions:**

A supervised multi-modal exercise training developed at primary care health units reduced the cardiovascular risk in adults living in very low-income communities. The risk remained stable in patients practicing self-directed physical activity and increased among individuals who remained physically inactive. These promising results should be considered within public health strategies to prevent cardiovascular disease in communities with limited resources.

**Trial registration:**

TCTR20181221002 (retrospectively registered). Registered December 21, 2018.

## Background

Cardiovascular diseases are the leading causes of death worldwide, including low and middle-income countries [1, 2]. The efficacy of regular physical activity to reduce cardiovascular risk has been widely accepted [3, 4]. In Brazil, the “Strategic Action Plan to Combat Chronic Non-Communicable Diseases” [1] launched by the Brazilian Health Ministry established that increased levels of physical activity should be one of the main goals to be attained between up to 2022. However, there is still need to develop and refine approaches to foster physically active behaviors in private and public health sectors [5, 6].

Recently, the American Heart Association urged healthcare systems and other stakeholders to promote physical activity in healthcare settings, in order to contribute to the prevention of epigenetic risk factors for non-communicable chronic diseases [7]. Evidently, this is more problematic in low-income countries, and particularly in communities with high social vulnerability. Although studies about the adherence to physical activity in low-income Brazilian communities are scarce [8, 9], there is evidence reinforcing that social vulnerability represents a major obstacle to the adoption of physically active behaviors [10]. In fact, citizens that dwell in those communities face greater obstacles to occupy their free time with physical activities, such as inadequate space for practice, poor time management, or unsafe environment [10].

The ‘Carioca Academy Program’ (CAP) has been created to provide opportunities for health-oriented exercise practice in socially vulnerable communities [11, 12]. The CAP is a community-based program of physical activities developed in Rio de Janeiro City (RJ, Brazil), which integrates the national program offering primary care services called ‘Family Health Strategy’ [13]. The major focus of the ‘Family Health Strategy’ is to develop primary health actions to prevent diseases associated with high morbidity and mortality, therefore reducing hospitalization rates [13, 14]. Moreover, it bestows opportunities for the development of interdisciplinary actions to promote health interventions within regions of low socioeconomic status, including physical activity [12, 15].

Physical activities provided by the CAP are considered as part of primary care actions and services, considering the current socio-demographic, epidemiological and nutritional characteristics of the served populations [12]. Supervised exercise programs are offered at primary care health units, in social spaces of high clinical and social vulnerability. Exercise modalities include gymnastics, dance, martial arts, resistance exercises, and hidrogymnastics [12, 16]. Evidently, the strategies applied by the ‘Family Health Strategy’ must be evaluated in order to optimize its interventions. In this context, studies about the effects of exercise routines applied by the CAP are warranted, and one of the major outcomes is the cardiovascular risk.

Official reference guides of primary prevention in Brazil recommend the use of the Framingham Risk Score (FRS) to assess the overall cardiovascular risk [17–19]. The risk estimation obtained from the FRS integrates data of age, total cholesterol, HDL-C, systolic blood pressure, current smoking, and diabetes, and the use of antihypertensive medication [20]. Despite these official recommendations, the few Brazilian studies addressing the cardiovascular risk using the FRS included very specific and small groups, as climacteric women [21] or hypertensive patients [22, 23]. The promotion of physical activity in community levels should contribute to lower the overall cardiovascular risk [24]. Therefore, it would be important to determine whether exercise routines proposed by the CAP contribute to achieving this goal.

Thus, the purpose of this study was to compare the cardiovascular risk estimated by the FRS in three groups attended over 12 months by the ‘Family Health Strategy’ at low-income communities in Rio de Janeiro City, including participants of supervised exercise training within the CAP, declared active individuals (self-directed), and physically inactive controls. We hypothesized that the reduction in FRS would be greater in patients that participated of supervised training in the CAP vs. those who declared to perform self-directed physical activities or to be physically inactive.

## Methods

### Participants

This longitudinal non-randomized controlled trial complied to the CONSORT Statement recommendations [25]. Patients of both sexes followed by the ‘Family Health Strategy’ in low-income communities (known as ‘*favelas*’) – *Mangueira* and *Tuiuti* – at Rio de Janeiro City were included in the study. Mangueira and Tuiuti are representative of socially vulnerable communities in Rio de Janeiro. They are geographically very close, consisting of what is called “favela complex”. These communities exhibit similar Social Development Indices (IDS) to other vulnerable communities in the city and its metropolitan area (known as “Grande Rio”) [26, 27]. In brief, Mangueira and Tuiuti were chosen because of their poor social indicators, high violence rates, and the fact that they count with well-structured Family Health Strategy units with fully operating CAPs. This was consistent with the purpose to demonstrate that even in a context of high social vulnerability it would be possible to promote health at public health facilities through supervised physical exercise.

In order to select individuals for the study, the electronic medical recordings of all patients aged 30- to 74 years (corresponding to FRS scope) were screened to identify those with complete information needed for risk stratification. The Family Health Strategy units in the observed communities had 19,780 registered patients, but only 1985 electronic records initially qualified. Of these, we were able to contact 1342 patients who were invited to participate in the study.

All patients were given the opportunity to enroll in the supervised exercise program offered by the CAP. Those who did not accept were invited to participate of one of the two control groups, according to their physical activity designation. In short, three groups were defined: 1) Formally Active (intervention group, FA), composed by individuals willing to participate of the supervised exercise program within the CAP three times a week; 2) Declared Active (active controls, DA), composed by individuals not formally engaged in supervised exercise, but having declared to perform physical activities in the free time, at least twice a week over the experimental period; 3) Physically Inactive (inactive controls, PI), composed of those who declared not to have practiced physical activities over the experimental period.

Patients should meet the following inclusion criteria for eligibility: a) free from heart disease or stroke; b) without clinical manifestations of atherosclerosis or dyslipidemias of genetic origin, c) not be engaged in supervised exercise programs over the 12 months preceding the study. Exclusion criteria were: a) to fail data collection for any reason; b) FA: poor adhesion to the supervised training program (frequency < 75% of planned sessions); DA: discontinuation of physical activity practice (at least twice a week) during the experiment. Physical activity and inclusion criteria information were obtained from medical recordings and direct interview with the physician. In all cases, individual screening for eligibility in FA occurred within 2 weeks prior to the beginning of supervised training.

**Fig. 1:**
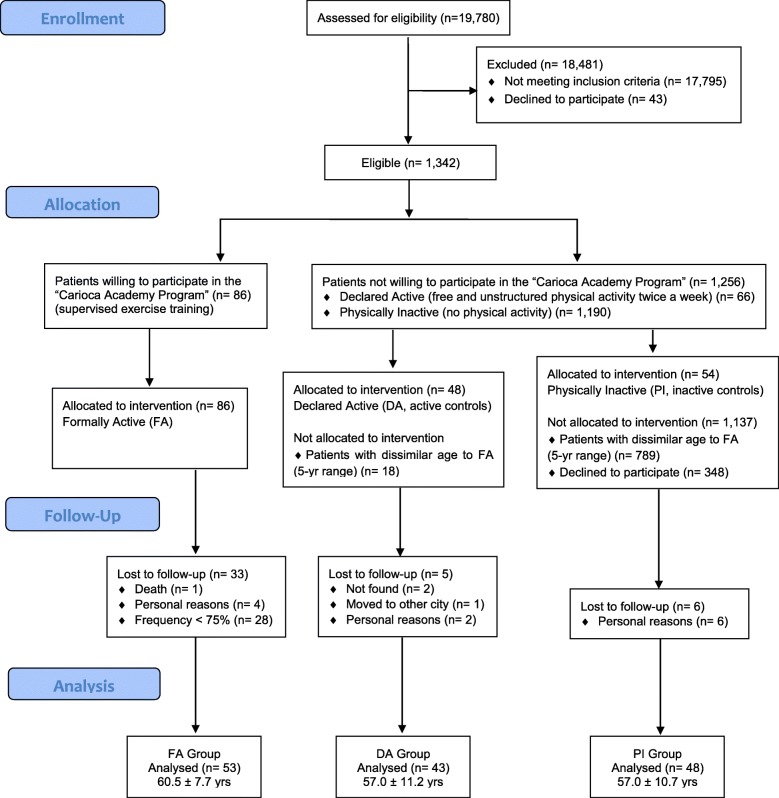
Sample selection and randomization into experimental groups

Figure 1 illustrates the sample selection and procedures for group randomization. Those who accepted to engage in the supervised exercise program (*n* = 86) were assigned into the intervention group (formally active; FA). Of these, five dropped out (1 death) during the experiment and 28 had frequency lower than 75% of exercise sessions. Therefore, 53 patients (60.5 ± 7.7 years) in FA completed the training program, producing a dropout rate of 38.4%. No dropout was due to injuries or health problems related to the exercise routine. The main reasons were unjustified low frequency (< 75%) to the planned exercise sessions (85%) and personal issues (12%). Only 48 patients with similar age to FA (5-year range accepted) declared to practice physical activities at least twice a week, being therefore assigned to DA. Of these, 43 patients (57.0 ± 11.2 years) returned to be reevaluated (5 patients not found, moved to other cities, or dropped out for personal reasons). Finally, the investigated Family Health Strategy units had 1208 physically inactive patients. Of these, 348 had similar age to FA and DA, but only 48 accepted to participate in the study and attended to all experimental procedures (PI group, 57.0 ± 10.7 years).

### Experimental design

As aforementioned, potentially eligible patients were initially contacted by telephone, being informed about the research and invited to attend the basic health unit after a 12-h fast. On the first visit, all volunteers were invited to join the CAP. Doubts about the research were clarified and those willing to participate signed informed consents. Subsequently, participants underwent an interview to the determination of the clinical and demographic features, blood pressure and anthropometric measurements, and venous blood collection.

Individuals assigned to FA participated in a supervised physical exercise intervention in the CAP for 12 months. The training routine included 10 strength and aerobic individual exercises that were performed in specific days twice a week in devices installed at the Family Health Strategy units. The intensity and workload progression were established based on the perceived exertion, ranging from moderate to vigorous. The number of sets in each exercise ranged from 2 to 4, depending on the individual physical conditioning. The exercises were ordered in a circuit format with 30–60 s rest intervals between sets and exercises, with a total duration of 50 min. Before the exercise circuit, a 5-min warm-up was applied and stretching exercises were performed as cool-down in the last 5 min of training sessions. Additionally, the participants were asked to perform, in another specific day, a brisk walk with duration of 30–60 min, totalizing three sessions a week of physical exercises. This activity was performed in groups, and to assure the minimum duration of 30-min it was monitored by a professional from the basic health unit (not necessarily an exercise specialist). This amount of exercise complied with recommendations of health agencies in regards to exercise prescription to promote health [28].

Since the number of dropouts in FA was considered relatively high, an intention-to-treat approach was adopted to verify the effects of the supervised training program upon the cardiovascular risk. After the first evaluation, patients assigned to DA and PI were contacted every 2 months in order to confirm whether they maintained the regular practice of physical activities or remained physically inactive, respectively. After 12 months, all groups should return to the health unit to repeat blood pressure, anthropometric, and biochemical blood assessments. All measurements were performed by trained physicians and nurses according to standard protocols applied at the basic health unit.

### Procedures

Demographic information (age, sex, ethnicity, education, and work) was obtained by a structured interview. In addition, participants were asked about their habits regarding smoking and physical activities (frequency, intensity, duration, and modality). Body mass and height were measured by a mechanical balance with a stadiometer (W300 A, Welmy™, Sao Paulo, SP, Brazil) according to standardized procedures. Diabetes and hypertension were identified through information from electronic medical records.

Biochemical blood variables (total cholesterol, LDL-C, HDL-C, triglycerides, and glycosylated hemoglobin) were determined by standard enzymatic methods after 12-h fasting. Blood pressure was measured on the left arm using a mercury column sphygmomanometer (Accumed-Glicomed™, São Paulo, SP, Brazil). After 5 min of seated rest, the average of three consecutive measurements with 2-min intervals between each one was recorded.

The FRS was calculated by means of a public worksheet available on the Framingham Heart Study website (www.framinghamheartstudy.org). In short, the variables sex, age, systolic blood pressure, presence of hypertension and diabetes, smoking, HDL-cholesterol and total cholesterol are included in an algorithm for the cardiovascular risk estimation. The risk for cardiovascular events in the next 10 years is classified as low (≤ 6%), moderate (≥ 6% and ≤ 20%), or high (> 20%).

### Statistical analyzes

Data normality was confirmed by the Shapiro-Wilk test. General estimating equations (GEE) according to the distribution of each dependent variable were applied for comparisons between groups at baseline (crude analysis) and along time (adjusted by covariates sex, chronological age, body mass index, and the use of anti-hypertensive medication or statins). Post-hoc statistical power was calculated based on estimated effect size (medium), sample size, number of groups, time-points, and covariates. Main analysis adopted an intention-to-treat approach, including participants that completed the supervised training and those with frequency lower than 75% of the sessions (*n* = 28). Estimated marginal means and 95% confidence intervals were also calculated. Statistical significance was set at *P* < 0.05 and all analyses were performed using the STATA 15.1 software (StataCorp™, College Station, TX, USA).

## Results

Table 1 exhibits clinical and demographic characteristics of all groups. Individuals assigned to FA are exhibited in subgroups, those with frequency lower (dropouts) and higher than 75% of the training sessions (FA complete).

**Table 1:** Clinical and demographic characteristics of the sample at baseline

Variable	FA dropout (*n* = 28)	FA complete (*n* = 53)	DA (*n* = 43)	PI (*n* = 48)
Sex (%)				
Female	93	89	72	73
Male	7	11	28	27
Ethnicity (%)				
White	54	55	33	33
Non-white	46	45	67	67
Occupation (%)				
Not working	50	83	67	83
Working	50	17	33	17
Medication (%)				
Betablocker	29	21	5	25
Antihypertensive	46	76	72	71
Hypoglycemiant	39	34	9	31
Statin	46	38	26	29
Clinical Status (%)				
Diabetes	39	30	14	26
Hypertension	46	77	70	67
Smoking	7	6	2	2
Overweight	21	36	40	27
Obesity	60	47	49	54

*FA* formally active (intervention group), *DA* active controls (self-directed physical activity), *PI* inactive controls

Overall, participants were predominantly women (82%) and non-white (56%). Approximately 31% of individuals were overweight and 53% were obese. In which concerns the socioeconomic status, most participants were retired or unemployed. In fact, 71% of participants had no formal labor activity. The working rate was higher among dropouts. The prevalence of hypertension was lower in the subgroup ‘FA dropout’ (46%) vs. other groups (67- to 77%), while more than 90% of patients did not smoke. The presence of diabetes was lower in DA (14%) than in FA (30–39%) or PI (26%). Consistent with these findings, the use of oral hypoglycemic agents was also lower in DA (9%) vs. FA (34–39%) or PI (31%), while less than 50% of FA dropouts used anti-hypertensive medications vs. more than 70% of patients in the other groups. The use of statins was similar across groups, while DA had considerably lower utilization of beta-blockers in comparison with FA (21–29%), DA (5%) or PI (25%).

Table 2 summarizes data included in the FRS calculation at baseline in all groups. FA includes patients that completed the training sessions and dropouts, according to the intention-to-treat approach. No difference between groups was detected for any of the observed variables (*P* > 0.05).

**Table 2:** Characteristics of the sample at baseline according to experimental groups

	FA (*n* = 81)	DA (*n* = 43)	PI (*n* = 48)	*p-*value
Chronological age, years	59.4 ± 7.6	57.0 ± 11.2	57.0 ± 10.7	0.245
BMI, kg/m^2^	30.2 ± 5.8	31.0 ± 6.2	30.5 ± 6.2	0.806
Total cholesterol, mg/Dl	204.9 ± 43.7	199.6 ± 38.6	201.3 ± 54.9	0.065
HDL-C, mg/dL	50.6 ± 13.3	49.3 ± 12.1	46.6 ± 13.8	0.270
LDL-C, mg/dL	126.3 ± 37.8	123.7 ± 34.7	121.8 ± 52.0	0.190
Triglycerides, mg/dL	141.6 ± 55.1	133.0 ± 59.0	164.9 ± 95.0	0.651
SBP, mmHg	132.2 ± 17.0	135.4 ± 15.8	131.9 ± 17.8	0.401
DBP, mmHg	82.8 ± 10.0	84.7 ± 6.7	84.8 ± 7.7	0.373
Framingham Risk Score	0.16 ± 0.12	0.14 ± 0.13	0.16 ± 0.12	0.134

*Note. FA* formally active (intervention group), *DA* active controls (self-directed physical activity), *PI* inactive controls. *BMI* body mass index, *HDL-C* high density lipoprotein – cholesterol, *HDL-C* low density lipoprotein – cholesterol, *SBP* systolic blood pressure, *DBP* diastolic blood pressure. Data are reported as mean ± standard deviation

Table 3 presents the characteristics of exercises performed by FA (e.g., individuals with frequency > 75% of sessions) and DA (frequency, intensity, session duration, and type of exercises). In general, the frequency was lower among individuals assigned to DA, which predominantly practiced physical activities twice a week. In contrast, the intensity was higher in FA, being predominantly moderate.

**Table 3:** Classification of exercises performed by formally active (FA) and declared active (DA) groups according to training variables

	FA (*n* = 53)	DA (*n* = 43)
Frequency (sessions/week)		
2	–	51%
3	100%	39%
> 3	–	10%
Intensity^a^		
Light (Borg 1-3)	14%	69%
Moderate (Borg 5-6)	63%	31%
Vigorous (Borg 7-8)	23%	0%
Time (min)		
30–60 min	–	75%
60–90 min	100%	15%
> 90 min	–	10%
Type (modalities)		
Walking	NA	13%
Jogging	NA	77%
Calisthenics/Functional	NA	10%

*Note.* ^a^Intensity estimated by means of the Borg CR-10 Scale (0–10), *NA* not applicable

Table 4 depicts data from intention-to-treat analysis, by means of adjusted GEE of the effect of different interventions on lipid profile and blood pressure. Even including the dropouts, all risk markers decreased in FA over time (*P* < 0.05), except the HDL-C which did not change from baseline (*P* > 0.05). In DA, only LDL-C lowered after the intervention (*P* < 0.05). Post hoc achieved power analysis (1 – β) revealed that our sample size was not adequate only for HDL-C analysis. On the other hand, PI (or controls) increased their total cholesterol, LDL-C, TGL, and systolic blood pressure (*P* < 0.05). In consequence, at the end of the experiment, FA exhibited lower total cholesterol, LDL-C, TGL, and blood pressure (systolic and diastolic) vs. PI (*P* < 0.05). Moreover, systolic and diastolic blood pressures became significantly lower in FA vs. DA (*P* < 0.05).

**Table 4:** Adjusted generalized estimating equations of the effect of different interventions in lipid profile and blood pressure

Variable	FA (*n* = 81)		DA (*n* = 34)		PI (*n* = 48)		*p-*value	Power (1-β)
	Baseline	Follow-up	Baseline	Follow-up	Baseline	Follow-up	Interaction	
Total cholesterol, mg/dL	202.3 (192.9 to 212.2)	187.3*^a^ **(178.1 to 197.0)**	200.7 (189.4 to 212.8)	193.0 (180.3 to 206.6)	183.1 (168.7 to 198.7)	206.6* (188.9 to 225.8)	**0.002**	0.91
HDL-C, mg/dL	49.7 (47.1 to 52.5)	50.4 (47.8 to 53.1)	50.2 (46.7 to 53.9)	49.5 (45.8 to 53.6)	47.3 (43.5 to 51.4)	45.5 (41.9 to 49.5)	0.537	0.21
LDL-C, mg/dL	124.9 (116.9 to 133.4)	111.1*^a^ **(103.8 to 118.9)**	124.3 (114.1 to 135.3)	113.2* (101.6 to 126.2)	112.3 (101.0 to 124.9)	128.8* (115.6 to 143.6)	**0.001**	0.86
Triglycerides, mg/dL	145.6 (130.0 to 154.3)	124.8*^a^ **(114.6 to 135.9)**	133.3 (116.9 to 152.0)	141.0 (121.8 to 163.1)	131.5 (115.7 to 149.4)	158.2* (137.2 to 182.2)	**0.001**	0.99
PAS, mmHg	131.9 (128.5 to 135.3)	114.0*^ab^ **(111.2 to 116.8)**	135.6 (131.1 to 140.3)	134.4 (129.4 to 139.5)	132.2 (127.5 to 137.1)	141.4* (136.0 to 147.0)	**< 0.001**	1.00
PAD, mmHg	82.9 (80.8 to 84.9)	77.7*^ab^ **(76.3 to 79.2)**	84.6 (82.6 to 86.6)	82.8 (79.5 to 86.3)	84.8 (82.8 to 87.0)	86.2 (83.6 to 88.9)	**< 0.001**	0.83

*FA* formally active group, *DA* declared active group, *PI* physically inactive group. *BMI* body mass index, *HDL-C* high density lipoprotein – cholesterol, *LDL-C* low density lipoprotein – cholesterol, *SBP* systolic blood pressure, *DBP* diastolic blood pressure. *Note*: Intention-to-treat: FA includes individuals that completed the supervised training (*n* = 53) and dropouts (*n* = 28). Interaction refers to the interaction group vs. time. Analyses adjusted for sex, chronological age, body mass index and hypertensive/statin drugs ingestion. Values presented through estimated marginal means (95% confidence interval).

(Bold data) *P < 0.05 vs. Baseline, ^a^*P* < 0.05 vs. PI, ^b^*P* < 0.05 vs. DA

**Fig. 2:**
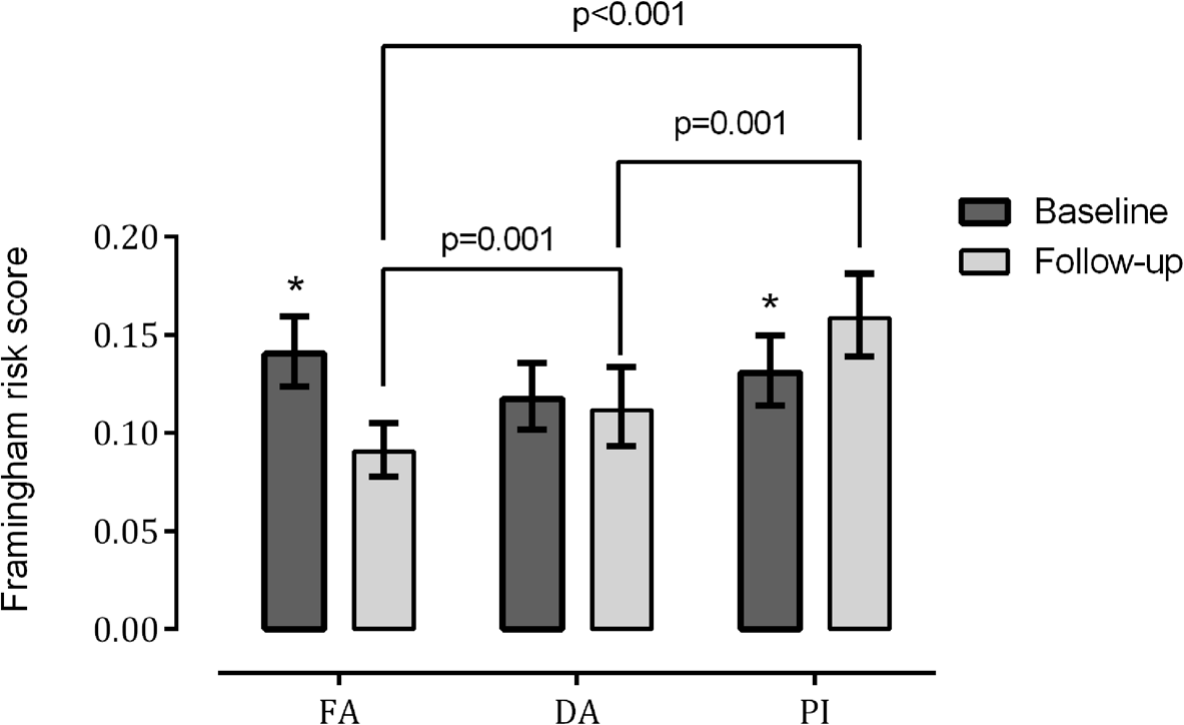
Adjusted generalized estimating equations of the effect of different interventions upon the Framingham risk score. FA: intervention group, including patients that completed the supervised training (*n* = 53) and dropouts (*n* = 28) (intention-to-treat approach); DA: active controls, self-directed physical activity performed twice a week (*n* = 43); PI: inactive controls, patients remained physically inactive throughout the experiment. Solid lines indicate significant differences between groups. *: *P* < 0.05 vs. baseline. Analyzes adjusted for sex, chronological age, body mass index and hypertensive/statin drugs ingestion. Power (1-beta): 1.00

Finally, Fig. 2 presents data for adjusted GEE of the effects of interventions upon FRS. The intention-to-treat analysis revealed that overall cardiovascular risk lowered approximately 35% in FA, remaining stable in DA, and increasing by 20% in PI. In consequence, after the 12-month intervention, the FRS became significantly lower in FA vs. DA and PI (*P* < 0.05).

## Discussion

This study has shown that the exercise training performed at Family Health Strategy units was able to reduce the overall cardiovascular risk of previously inactive individuals, by improving blood pressure, body composition, and biochemical blood markers. The FRS remained stable among those who declared to perform self-directed physical activities over 12 months, and increased in physically inactive patients. Therefore, we confirmed the hypothesis that the supervised training applied within the CAP was more effective to reduce the cardiovascular risk over 12 months than unstructured and free physical activity.

The intervention program provoked favorable changes in several cardiovascular risk markers, especially blood lipid profile, blood pressure, and type-2 diabetes [29–31]. Since those factors integrate the FRS calculation, the lower cardiovascular risk found in DA and FA vs. PA was thereby expected. However, this was particularly true for patients who underwent supervised training (FA). Even including the dropouts in the analysis, the reduction in cardiovascular risk was markedly greater in FA than in active controls (DA). Actually, 5 of 6 cardiovascular risk markers improved in FA, while only LDL-C reduced in DA (see Table 4). There is evidence suggesting that interventions conducted within the Family Health Strategy may increase the physical activity levels of participants. Ribeiro et al. [32], for instance, conducted a 12-month non-randomized trial investigating the effect of physical activity and health education interventions on the levels of physical activity of users of Brazilian Unified Health System attended by the Family Health Strategy, in the city of Sao Paulo (SP, southeastern Brazil). Both groups increased their overall time of physical activity (physical exercise, leisure, and transport-related), but individuals assigned to the health education group had a greater tendency to maintain a physically active routine than those who only attended exercise classes.

In the present study, the cardiovascular risk estimated by the FRS increased in PI and remained stable in DA, which means that in terms of the prevention of cardiovascular diseases, self-directed physical activity would be better than no physical activity at all. The amount of exercise in DA seemed to be enough to prevent the worsening of those factors, which explains the stability of FRS throughout the experiment. It is worthy to comment why the self-reported exercisers did not look different vs. FA and DA, which were previously inactive at the occasion of initial screening (please refer to Table 2). We can speculate that the intensity and volume of self-directed and unstructured physical activities performed by DA did not meet the minimum requirements to induce changes in outcomes related to the cardiovascular risk. However, this seemed to be enough to stabilize their condition – perhaps this was also the case before the experiment. In contrast, our results reinforce the idea that supervised physical training, even when simple and inexpensive, might have greater benefits than unstructured activities in which concerns the prevention of cardiovascular diseases. The intention-to-treat approach is conservative in the production of significant effects of experimental interventions. Therefore, our results are promising and deserve consideration within public health promotion policies, particularly in developing countries.

Our findings are consistent with the premise that active lifestyles contribute to lower risk of developing cardiovascular diseases [33]. This trial contributes to the current knowledge by demonstrating that strategies that contemplate supervised exercise programs within public health units can induce a greater reduction in cardiovascular risk than only stimulating the free practice of physical activities. The association between physical inactivity and increased cardiovascular risk as estimated by FRS has been previously demonstrated. Galvão et al. [34] reported that a physically inactive lifestyle was more prevalent in men with FRS corresponding to high risk vs. low to moderate risk. Silva et al. [35] revealed that 83.3% of a postmenopausal women sample was classified as active or very active, whereas 87.7% obtained FRS scores corresponding to low cardiovascular risk the physical activity level. In which concerns the ‘Family Health Strategy’, we could find a single trial investigating the effects of a 20-week supervised exercise program on body composition and FRS [36]. Although the sample has been restricted to postmenopausal obese women, similarly to our intervention there was a significant improvement in triglycerides and systolic blood pressure, as well as a reduction in FRS score.

It should be highlighted that we recognize the importance of physical activities performed in the free time. A prior study from our laboratory has demonstrated that the only presence of physical activities during leisure can significantly reduce the risk for cardiovascular and metabolic diseases [37]. However, our findings suggest that this will not always achieve certain goals. It is feasible to speculate that differences in risk evolution between FA and DA were due to the fact that only FA met the minimum recommendations of health agencies for exercise prescription aiming at health promotion [38]. In general, recommendations for physical training to promote health usually include aerobic exercises performed 3- to 5 days a week, with a duration of at least 30 min and moderate- to vigorous intensity. Moreover, recommendations also include a minimum of 1 set of 10 to 15 repetitions for 8 to 10 resistance exercises involving the major muscle groups, as well as flexibility and balance exercises at least 2 days a week [38].

It is usually accepted that dose-response relationships regarding several cardiovascular risk factors increase with exercise intensity and volume [28, 38]. Interestingly, 69% of patients assigned to DA classified their physical activities as ‘light’, which reinforces the premise that these patients did not perform a sufficient amount of exercise to induce significant changes of cardiovascular risk factors included in FRS calculation. Our results support the importance of supervised programs to ensure adequate exercise prescription for health promotion. An adequate control of training stimuli within exercise programs developed in public health units seems to be possible, which warrants attention from public health managers.

Similarly to other intervention studies with physical activity applied within the Family Health Strategy [10, 32], a classical randomization of participants in experimental and control groups was not possible in the present study. Firstly, only a small segment of the initially contacted individuals agreed to participate of the supervised exercise program. Moreover, we could not simply dismiss part of volunteers by assigning them to a control group, due to ethical problems – we could lose them, and this was not acceptable. It must be noticed that this experiment was carried out with minimum interference in the routine of the Family Health Strategy units. For this reason, there was no change in the procedures usually adopted by the CAP to recruit patients for the supervised exercise program.

The dropout rate among the patients that enrolled in the physical training was of approximately 38%, which is undeniably high. However, this rate was similar to values reported by studies developed in different countries that applied exercise interventions in high social vulnerability contexts [10, 39]. Overall, adherence remains a challenge to implement exercise routines within the Family Health Strategy. Despite wide advertising prior to the beginning of the program, the reach was low; of the initially contacted 1342 patients, only 86 accepted to participate of the supervised training and 53 completed the 12-month intervention. This difficulty has been reported in other studies developed in Brazilian socially vulnerable communities [40].

Although recognizing that the admission was limited considering the total population, we cannot say that the CAP has low acceptability or poor choice for the overall population. Actually, over 130,000 participants attend the program, which is present in 202 out of 231 health units in social vulnerable areas, including patients of all ages [11, 16]. The differential of the CAP program relates not only to the characteristics of the exercise intervention (volume, intensity, modalities etc.), but also to the fact that social environment is considered in its scope. The type of activities and approach to patients takes into account geographic (availability of public facilities) and social (violence, presence of drug traffic, age profile etc.) features of the areas where the program is inserted, which allows reaching different populations and groups. Hence, although focusing on priority target audiences (e.g., patients with non-communicable chronic diseases and the elderly), the CAP does not exclude other users [12]. Finally, since this is an exercise program linked to other health services, the patient remains close to the health unit which makes easier a constant monitoring.

In the specific case of our study, when we say that only 86 of the eligible 1342 patients agreed to enter the exercise program, we have to consider the profile of the investigated communities. Most of patients registered at the health unit were young adults that studied or worked in the hours the CAP program was offered. This helps to explain the relatively high average age of the participants in the study (around 60 years-old). Even considering this is the age-range that could benefit more of a lowered cardiovascular risk, this is perhaps the greatest challenge of the CAP in the future – how to provide options for individuals that cannot exercise during typical working hours, in communities where night activities are precluded due to high violence rates. As mentioned in the manuscript, the dropout rate of 38.4% also relates to these features, which is consistent with the difficulties to select patients for 1-year follow-up.

As abovementioned, a large proportion of residents studied or worked full-time. Hence, dwellers in those communities seem to be more prone to physical inactivity in comparison with those who inhabit wealthier spaces, due to geographic, social, and economic factors [8]. In Brazil, Galvim et al. [10] analyzed the adherence, adhesion, and dropout reasons to a 6-month guided walk program offered to 106 patients attended by Family Health units in the city of São Carlos, SP, Brazil. The dropout rate was of approximately 50% and the main reported reasons were the working hours (28%), health (26%) and personal reasons (22%), or lack of time (11%). Those factors might also explain the high number of dropouts observed in our study, especially in FA. As abovementioned, the rate of employment among dropouts was three times higher than in the group that completed the supervised training. Conversely, the dropout rate in DA was of 10.4%. In this specific group, most individuals remained physically active during the 12-month intervention, perhaps due to the fact they were free to practice the chosen activities at their best convenience of time and local. Further studies are warranted to ratify this premise.

Randomized controlled trials frequently suffer from two major complications, i.e., noncompliance and missing outcomes. In order to avoid bias due to excessive dropouts, a potential solution to this problem is the intention-to-treat concept. The dropout rate within patients that underwent the supervised training was of almost 40%, which was considered high enough to introduce bias in the results. Therefore, we decided to analyze the outcomes using the intention-to-treat approach [41]. In short, data from all patients initially assigned to FA were included in the statistical analysis, despite the treatment they actually received, and regardless of withdrawal from the exercise routine. This strategy avoids overoptimistic estimates of the efficacy of a given intervention resulting from the removal of non-compliers, by accepting that noncompliance deviations are likely to occur in actual clinical practice [41].

Unfortunately, we did not assess the physical activity level of the analyzed groups (FA, DA, and PI). The contacts established every 2 months were enough to confirm that no change in patients’ routine occurred. Therefore, those assigned in PI remained physically inactive, while changes in physical activity routine did not occur among in DA. This procedure was considered as enough to exclude or maintain the individuals in our experiment. Prior studies have suggested that sometimes the control groups increase their physical activity level after interviews about this topic [32, 40]. Even accepting that this effect might have occurred in DA and PI, this would not change the fact that physical activity would remain self-directed and unstructured in DA, while individuals in PI continued to declare that they were physically inactive. Thus, bias due to this possibility was unlikely to have occurred.

The major limitation of this study was that participants were allocated in the experimental groups according to their desire to participate or not of the supervised exercise program in the CAP. As explained above, a classical randomization in experimental and control groups was not possible, since we did not intend to interfere in the routine of the Family Health Strategy unit. Another potential limitation was that the Framingham cohort was based on data from a mostly white population of European origin. Although previous studies have demonstrated the applicability of the FRS in other populations [42–44], we have to acknowledge that this may have interfered with the sensitivity of our results (see http://www.framinghamheartstudy.org). In addition, the sample size was not sufficient to elicit adequate statistical power for comparisons regarding HDL-C. Finally, only a few low-income communities located in the North Zone of Rio de Janeiro City were included in the experiment. Additional trials including health units integrated to the CAP in other regions of the city should be encouraged.

## Conclusion

The supervised training provided by the CAPs is simple and inexpensive, therefore increasing the access of poorer groups to the practice of oriented physical activities. This type of strategy may be an option for health promotion in countries where resources are limited, which is the case of most developing countries. Our results suggest that a 12-month multimodal supervised exercise program developed at public health units can reduce cardiovascular risk as estimated by the FRS in adult patients living in very low-income communities. On the other hand, the cardiovascular risk remained stable in patients who declared to be regularly involved in self-directed physical activities, while increased in physically inactive individuals followed for the same time.

In order to ratify these findings, future studies should investigate the efficacy of different approaches to physical exercise in primary prevention of cardiovascular disease and health promotion, not only in Rio de Janeiro City but also in other health programs and sectors, particularly those attended by the public health system.

## Data Availability

The datasets used and/or analyzed during the current study are available from the corresponding author on reasonable request.

## Abbreviations

CAP: Carioca Academy Program
DA: Declared Active
FA: Formally Active
FITT: Frequency, intensity, time, and type of exercise
FRS: Framingham Risk Score
Hb1Ac: Glycosylated hemoglobin
HDL: High-density lipoprotein
LDL: Low-density lipoprotein
PI: Physically Inactive
TGL: Triglycerides
